# Role of Sensory Appeal, Nutritional Quality, Safety, and Health Determinants on Convenience Food Choice in an Academic Environment

**DOI:** 10.3390/foods10020345

**Published:** 2021-02-05

**Authors:** Hena Imtiyaz, Peeyush Soni, Vimolwan Yukongdi

**Affiliations:** 1 School of Environment, Resource and Development, Asian Institute of Technology, P.O. Box 4, Klong Luang, Pathumthani, Bangkok 12120, Thailand; st116566@ait.ac.th; 2 Agricultural and Food Engineering Department, Indian Institute of Technology, Kharagpur 721302, India; 3 School of Management, Asian Institute of Technology, P.O. Box 4, Klong Luang, Pathumthani, Bangkok 12120, Thailand; vyukongdi@ait.ac.th

**Keywords:** convenience food, determinants, purchase intention, consumption, confirmatory factor analysis, structural equation modeling

## Abstract

The present research aims to investigate the extent to which sensory appeal, nutritional quality, safety, and health determinants influence purchase intention, consumption, and satisfaction of consumers towards convenience food. The non-probability purposive sampling approach was adopted for the recruitment of consumers. A pre-tested questionnaire was used to collect data from 501 consumers. Descriptive statistics, confirmatory factor analysis, and structural equation modeling were adopted to analyze the data. Factor loading, Cronbach’s alpha, composite reliability, average variance extracted, and correlations estimate of constructs revealed good internal consistency and reliability of scale items as well as convergent and discriminant validity of the constructs. The path analysis of structural model demonstrated positive relationship between sensory appeal, nutritional quality, safety attributes, healthiness, and purchase intention of convenience food. Further, the path analysis of structural model revealed that purchase intention with consumption as well as consumption with satisfaction were positively associated for convenience food. Sensory appeal was the key determinant influencing purchase intention, consumption, and satisfaction of consumers towards convenience food. The good taste, pleasant appearance, nice smell, and appealing texture within sensory appeal were the most important factors influencing purchase intention, consumption, and satisfaction of consumers towards convenience food. Further, the consumers in emerging economies such as India focus more on sensory appeal in convenience food choice.

## 1. Introduction

Busy and hectic lifestyles, increase in working population and urbanization, increase in per capita and disposable incomes, diminishing trend of cooking skills and motivation, the rapid expansion of convenience food retail chains, significant improvements in food processing and packaging technologies, and significant change in food-related lifestyles have increased the demand and consumption of convenience food in both developed and emerging economies [1,2]. The global, United States, and European convenience food market is anticipated to rise at the compounded annual growth rate (CAGR) of 4.49%, 4.2%, and 4.5% respectively during 2020 to 2025. The convenience food market in the Asia Pacific region is expected to grow at a CAGR of 8.79% during 2020–2025. The Indian convenience food market generated a revenue of USD 261 million in 2017. It is anticipated to grow at a CAGR of 16.24% during 2019–2024 and reach a revenue of USD 931 million in 2024 [1]. The key market players of convenience food in India are Nestle, ITC, MTR, Capital Foods, CG Food, Haldiram, Bambino, GITS, Kohinoor, Kitchens of India, Maiyas, and Vshodaya [2].

Sensory characteristics such as taste, appearance, freshness, texture, color, and smell are essential motivating factors, driving consumers towards shopping and consumption of convenience food products. Due to advances in food processing and packaging technology, the sensory appeal of convenience food products has been considerably improved in recent years. The sensory appeals undoubtedly are believed to influence consumers’ perception, purchase intention, consumption, and satisfaction towards convenience food products significantly [3,4,5,6,7,8,9,10]. Prescott et al. [11] revealed that convenience, sensory appeal, quality, safety, price, and health are the important determinants influencing consumer shopping and consumption of convenience food. However, the magnitude and importance of each determinant may vary across Japan, Taiwan, Malaysia, and New Zealand. Wang et al. [12] revealed that sensory attributes, particularly taste, were the most important motivating factors positively associated with consumers purchase intention of traditional and Western convenience food in mainland China. The quality of convenience food products also drives consumers towards its purchase and consumption. Therefore, it is directly linked to the consumers’ perception, purchase decision, and consumption behavior [13,14,15,16,17,18]. The food quality certification from authorized agencies and brands provide the details of production process, ingredients, nutritional facts, shelf life, cooking instruction, place of production, sensory appeal, quality, safety, and environmental issues which are the primary concerns of consumers while purchasing convenience food [19]. Ojha et al. [20] revealed that high-pressure processing (HPP), pulse UV light, and irradiation technologies should be adopted to enhance sensory appeal, quality, and safety of convenience food products.

Food safety, another important determinant, influences the shopping and consumption of convenience food products. The consumers usually expect that the government food regulatory authorities, food processing industries, and marketing agencies take responsibility for the safety of convenience food products. Food safety is one of the most influential factors in terms of shopping and consumption of convenience food products [21,22,23]. The primary concerns of consumers about food safety are chemical, microbiological, and technological issues as well as the place of origin/place of products [24]. Vital demographic characteristics such as age, gender, education, marital status, and employment status considerably influence the food safety knowledge and practices [25,26]. Misra et al. [27] revealed that application of novel food processing technologies reduced processing time and energy consumption as well as assured high food safety of convenience food products. Health is one of the prime concerns of consumers while purchasing and consuming convenience food products. It is generally believed that regular and excessive use of convenience food causes obesity and other health-related problems [28,29]. Health is a multidimensional construct that influences the purchase intention and consumption of convenience food [4,30,31]. Hoek et al. [32] stated that the government regulatory authorities, responsible for the formulation of food laws and regulations, should prioritize health and health-related attributes of convenience food.

Socio-demographic trends in emerging economies have recently been indicating a major shift. These include more educated and entrepreneur youth population residing in megacities with increased proportion of monthly income on food, lack of time to spend on cooking, multiple income family, and above all the dynamic lifestyle. The confluence of these driving vectors leads to a forthcoming sprawl of convenience food. Several studies in the recent past have been carried out to seek the effect of various factors on convenience food choice, most of which focused on markets in developed and industrialized countries [4,7,8,9,23,26,31,33]. Due to diversity in tradition, culture, food habits, social structure, religious beliefs, and ethical values, the consumers in emerging economies might not respond to such factors in the same way as reported in aforementioned studies. Hence, it is important as well as timely to administer such research. Considering the impressive market growth and economic importance of convenience food in emerging economies such as India, the main goal of the study is to “examine the role of sensory appeal, nutritional quality, safety, and health determinants on purchase intention, consumption and satisfaction of consumers towards convenience food” in an academic environment.

## 2. Theoretical Background and Development of Hypotheses

### 2.1. Sensory Appeal

Sensory appeal plays a significant and important role on perception, purchase decision, consumption, and satisfaction of consumers towards convenience food [8,9,12]. Sensory characteristics such as taste [6,8,9]; flavor [8,34]; appearance [5,6]; freshness [5]; texture [6]; smell [6,8]; and overall liking [8] are important motivating factors driving consumers towards shopping and consumption of convenience food. Due to advances in food processing and packaging technologies, the sensory attributes have been improved considerably in recent years to motivate consumers towards convenience food choice [20]. Studies carried out in the past revealed that taste within sensory attributes was the key factor influencing consumer perception, purchase intention, and consumption of convenience food [8,9]. Considering the aforementioned research findings, the present study proposed the following hypothesis:

**Hypothesis** **1.** *Sensory appeal is positively related to purchase intention of convenience food*.

### 2.2. Nutritional Quality

Nutritional quality is another most important determinant, which motivates and drives consumers towards convenience food choice as well as being directly linked with perception, purchase decision, and consumption. Nutritional quality attributes such as nutritional value [15], natural ingredients [16], protein content [16], fiber content [16,17], vitamin content [17], mineral content [16], and nutritional quality certification [12] are the important factors, which drive consumers towards purchase intention and consumption of convenience food. Mascarello et al. [33] revealed that consumer’s positive perception towards quality attributes considerably influenced the purchase intention of convenience food. Based on the aforementioned research findings, the following hypothesis is proposed:

**Hypothesis** **2.** *Nutritional quality attribute is positively related to purchase intention of convenience food*.

### 2.3. Safety

Food safety is another important determinant that influences the purchase decision and consumption of convenience food. Most developed countries have stringent food safety regulation to safeguard the consumers. However, in developing countries like India, food safety regulation enforcement is still in the development stage. The safety attributes such as additives [21,35], pesticides [21,23], hormones [21], color [23], artificial ingredients [23], and safety certification [16] contribute significantly in purchase intention, consumption, and satisfaction of consumers towards convenience food. Based on the aforementioned research findings, the following hypothesis is proposed:

**Hypothesis** **3.** *Safety attribute is positively related to purchase intention of convenience food*.

### 2.4. Health

Health is the prime concern of consumers while purchasing and consuming convenience food. Health is a multidimensional construct that embodies overall wellbeing of consumers regarding physical, mental, and social aspects [30]. Health-related issues such as calories [36], fat [36], salt [37], sugar [37], and balanced diet [38,39] play important roles in influencing consumers for purchase intention, consumption, and satisfaction towards convenience food. Hoek et al. [32] stated that government regulatory authorities responsible for the formulation of laws and regulations should prioritize health and health attributes of convenience food. Based on the aforementioned research findings, the following hypothesis is proposed:

**Hypothesis** **4.** *Healthiness is positively related to purchase intention of convenience food*.

### 2.5. Purchase Intention, Consumption, and Satisfaction

The purchase intention of consumers towards convenience is a complex process, and it is governed by a wide range of determinants. However, the importance of each determinant, which drives consumers towards purchase intention of convenience food, depends on food-related attitude and behavior. The perceived value of products, which is directly associated with convenience, sensory appeal, nutritional quality, safety, health, and price, has a positive influence on consumers’ purchase intention for convenience food [40,41]. Apart from social, cultural, and economic determinants, convenience food consumption is also influenced by convenience, sensory appeal, nutritional quality attributes, safety attributes, healthiness, and price [30,42]. Consumer satisfaction is a strategic focus of consumer-oriented food industries and marketing agencies to retain and maintain the consumers for repeated purchase and consumption of their convenience food. Convenience [43], sensory appeal [9], nutritional quality [33], safety [23], healthiness [31], price [44], and physical wellbeing [45] are important attributes of convenience food, which lead to consumer satisfaction and loyalty. In light of the aforementioned research findings, the following hypotheses are proposed.

**Hypothesis** **5.** *Purchase intention is positively related to consumption of convenience food*.

**Hypothesis** **6.** *Consumption of convenience food is positively related to consumer satisfaction*.

**Figure 1:**
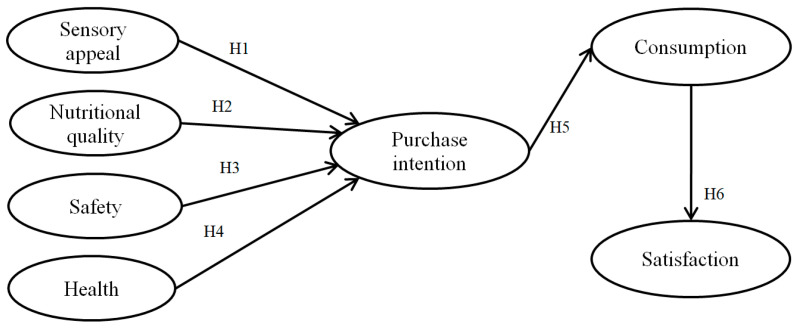
Conceptual model.

The conceptual model for the current study is based on aforementioned research findings to assess the role of sensory appeal, nutritional quality, safety, and health determinants influencing purchase intention, consumption, and satisfaction of consumer towards convenience food (Figure 1).

## 3. Materials and Methods

### 3.1. Development, Pre-Testing, and Structure of Questionnaire

A comprehensive literature review provided guidelines to develop a questionnaire to assess the role of sensory appeal, nutritional quality, safety, and health determinants on purchase intention, consumption, and satisfaction of consumers towards convenience food. Pre-testing is an important step to ensure the accuracy and reliability of the questionnaire [46]. The questionnaire was pre-tested at Sam Higginbottom University of Agriculture, Technology and Sciences, Allahabad, India to develop and optimize the questionnaire. The questionnaires were pre-tested with 30 participants comprising students, in-service professionals, and food processing and nutrition experts to identify and remove potential problems and ensure its comprehensibility. After completing the questionnaire, each participant was asked to give his/her feedback regarding clarity, comprehension, and potential problems to examine the role of sensory, appeal, nutritional quality, safety, and health determinants on purchase intention, consumption, and satisfaction of consumers towards convenience food. The suggestions made by the participants were included in the final questionnaire to ensure accuracy and precision in data collection [12,38,47,48].

The questionnaire was divided into eight sections. The first section was designed to collect general information of consumers such as socio-demographic characteristics, food habits, food preferences, frequency of eating convenience food, health concerns, etc. The second section of the questionnaire was designed to gather data regarding the sensory attributes (appearance, smell, texture, taste) influencing purchase intention of consumers of convenience food. The third, fourth, and fifth sections of the questionnaire were framed to collect data regarding nutritional quality (nutritive value, mineral, vitamin, natural ingredients, fiber, food quality certificate), safety (hormones, insecticides, pesticides, additives, food safety certificate), and health attributes (calories, fat, salt, sugar, balanced diet) influencing purchase intention of convenience food. The sixth, seventh, and eighth sections of the questionnaire were designed to collect data for purchase intention, consumption, and satisfaction of consumers towards convenience food (Appendix A).

### 3.2. Participants

The non-probability purposive sampling method was adopted for the recruitment of the participants because researchers were targeting a specific group of participants, i.e., university/college students, teaching and non-teaching staff, and professionals from corporate sector as they are the major consumers of convenience food consumption [47,49]. A total number of 550 participants were selected from four major cities of Northern India. The total population of four cities is approximately 8.25 million. The sample size of 550 participants taken in this study was higher than 400 as recommended over the population of 0.250 million with a confidence level of 95% and 5% margin of error [47,50]. A total number of 49 questionnaires were dropped due to incomplete information. The final sample size was 501, which resulted in a response rate of 91.09%.

### 3.3. Data Collection

The structured and pre-tested questionnaires were distributed to 550 participants in four universities, eight colleges, and twelve corporate offices in January 2019. The participants were requested to gather at the conference/meeting rooms provided by the universities, colleges, and corporate sectors. The participants were informed one day in advance regarding time and venue to achieve desired number of participants as well as to avoid inconvenience. A group of 25 participants were invited to complete the questionnaire. The researcher distributed the questionnaire to the participants and briefed them about purpose, objectives, and importance of the study. The influence of aforementioned determinants on purchase intention and consumption of convenience food were determined on five-point Likert scale (strongly disagree = 1, disagree = 2, don’t know = 3, agree = 4, strongly agree = 5). The participants were directed to choose one from 1 to 5 for each question [44,47,51].

### 3.4. Data Analysis

The statistical software SPSS version 24 was used to determine mean, standard deviation, skewness, and kurtosis. Further, SPSS was employed to determine Cronbach’s alpha to assess internal consistency and reliability of the scale items of questionnaire [47,52,53]. The AMOS software version 23 was used to perform confirmatory factor analysis (CFA) and structural equation modeling (SEM). The CFA was carried out to estimate factor loading, composite reliability, average variance extracted, and model fit indices. The composite reliability of the constructs of the questionnaire was determined to examine the reliability of scale items [38,47,48,52]. The factor loading and average variance extracted were determined to assess the convergent validity of the constructs of measurement model [12,38,39,48,52]. The correlations amongst the construct and square root of average variance extracted were used to examine the discriminant validity of constructs [54]. The statistical indices such as comparative fit index (CFI), Tucker–Lewis index (TLI), goodness of fit index (GFI), root mean square error of approximation (RMSEA), and standardized root mean-square residual (SRMR) were determined to examine the fit of measurement model [39,47,48,55]. The structural model was constructed to examine the association between sensory appeal, nutritional quality, safety, health, and purchase intention as well as purchase intention with consumption and consumption with satisfaction of consumers towards convenience food. The CFI, TLI, GFI, RMSEA, SRMR, and χ^2^/df (Chi square/degree of freedom) were determined to assess the fit of the structural model [47,48,53]. The standardized estimate (path coefficient), standard error, *t*-value, and *p*-value were determined to test the hypotheses [31,47,48].

## 4. Results

### 4.1. Descriptive Statistics

Table 1 demonstrates the socio-demographic characteristics of participants. The participants were students and teaching and non-teaching staff from universities/colleges and professionals from corporate sectors. The participants comprised of 41.3% males and 58.7% females with age ranging from 18–65 years (average age = 30.37). The participants consisted of 48.9% single and 51.1% married in which 34.1% and 65.9% were unemployed and employed, respectively. The participants’ education level ranged from high school to doctoral, i.e., high school (0.40%), senior secondary school (7.0%), diploma (1.4%), undergraduate (33.9%), master (34.5%), and doctoral (22.8%). The annual family income of the participants ranged from USD 700 to USD 40,000.

The mean participants’ score for sensory appeal was higher, followed by safety, nutritional quality, and health determinants influencing purchase intention, consumption, and satisfaction of consumers towards convenience food. The mean participants score of the items revealed that the “good taste” within sensory appeal construct; “food quality certification” within nutritional quality construct; “food safety certification” within safety construct; and “balanced diet” within health construct were the most important factors in relation to purchase intention, consumption, and satisfaction of consumers towards convenience food (Appendix A; Table 2). The skewness for different items of sensory appeal, nutritional quality, safety, health, purchase intention, consumption, and satisfaction were within the threshold value of −1 to 1. The kurtosis for different items of sensory appeal, nutritional quality, safety, health, purchase intention, consumption, and satisfaction fall within the acceptable range of −2 to 2 (Table 2). The skewness and kurtosis values indicated that participants’ score/data recorded for different items of sensory appeal, nutritional quality, safety, health, purchase intention, consumption, and satisfaction were normally distributed [4,53].

### 4.2. Measurement Model

The factor loading of all items of sensory appeal, nutritional quality, safety, health, purchase intention, consumption, and satisfaction for convenience food were significant (*p* ≤ 0.01). The factor loadings for different items of sensory appeal, nutritional quality, safety, health, purchase intention, consumption, and satisfaction constructs ranged from 0.608 to 0.963, which were higher than the threshold value of 0.50 [39,48,52,55], hence all items were included for the interpretation of the factors influencing purchase intention, consumption, and satisfaction of consumers towards convenience food [39,48,55]. Cronbach’s alpha for sensory appeal, nutritional quality, safety, health, purchase intention, consumption, and satisfaction constructs ranged from 0.740 to 0.897, which exceeded the threshold value of 0.70 [38,47,52]. Composite reliability for sensory appeal, nutritional quality, safety, health, purchase intention, consumption, and satisfaction constructs ranged from 0.852 to 0.979 that exceeded recommended minimum cut off value of 0.70 [47,48,52]. Cronbach’s alpha and composite reliability values obtained for different constructs revealed good internal consistency and reliability of scale items of questionnaire [3,48,54,55]. The average variance extracted for sensory appeal, nutritional quality, safety, health, purchase intention, consumption, and satisfaction constructs ranged from 0.521 to 0.864, which were higher than the minimum acceptable cut off value of 0.50 [39,48,54]. The factor loading and average variance extracted values obtained for different constructs and items for each construct confirmed the convergent validity of the constructs of measurement model [39,54,55]. The square root of average variance extracted estimates (diagonal values) were higher than the correlation estimates amongst constructs (Table 3), which confirmed the discriminant validity of constructs [47,48,54].

The comparative fit index (CFI), Tucker–Lewis index (TLI), goodness of fit index (GFI), root mean square error of approximation (RMSEA), and standardized root mean square residual (SRMR) were used to examine the fit of measurement model relating sensory appeal, nutritional quality, safety, and health aspects with purchase intention, consumption, and satisfaction towards convenience food. The CFI was 0.911 (≥0.90); TLI was 0.903 (≥0.90); GFI was 0.901 (≥0.90); RMSEA was 0.072 (≤0.08), and SRMR was 0.074 (≤0.08), which were within the acceptable range (Table 2). The CFI, TLI, GFI, RMSEA, and SRMR revealed that measurement model fit well with data [47,53,55,56].

### 4.3. Structural Model

**Figure 2:**
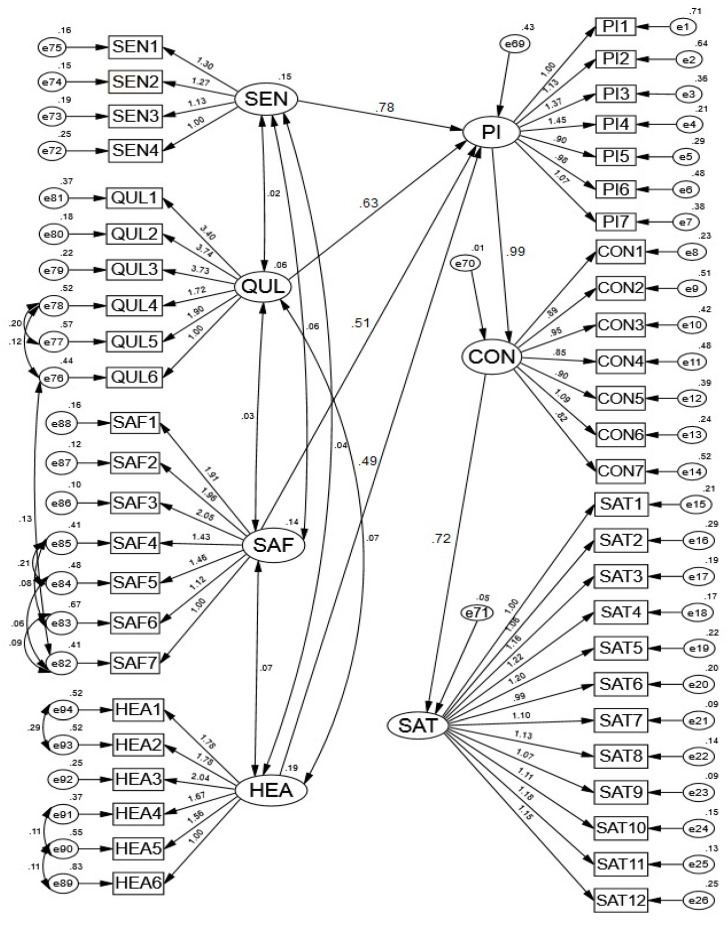
Structural equation modeling to assess the role of product determinants on purchase intention, consumption, and satisfaction of consumers towards convenience food. Structural model fit indices: CFI: 0.913; TLI: 0.906; GFI: 0.903; RMSEA: 0.073; SRMR: 0.075; χ^2^/df = 3.91.

The structural model was constructed to examine the association between sensory appeal, nutritional quality attributes, safety attributes, healthiness, and purchase intention as well as purchase intention with consumption and consumption with satisfaction of consumers towards convenience food. The CFI was 0.913 (≥0.90), TLI was 0.906 (≥0.90), GFI was 0.903 (≥0.90), RMSEA was 0.073 (≤0.08), SRMR was 0.075 (≤0.08), and χ^2^/df was 3.9 (<5.0), which were within the recommended acceptable range (Figure 2). The CFI, TLI, GFI, RMSEA, SRMR, and χ^2^/df values demonstrated a good fit of the structural model [39,47,53,56].

The results of the structural model presented in Figure 2 and Table 4 demonstrate the extent of the relationship among sensory appeal, nutritional quality attributes, safety attributes, healthiness, and purchase intention, as well as purchase intention with consumption and consumption with satisfaction for convenience food. Hypothesis 1 (H1), which proposed positive relationship between sensory appeal and purchase intention of convenience food was accepted, because standardized estimate (ß) of the path of structural model was significant (Hypothesis 1: ß = 0.788, S.E. = 0.053, *t*-value = 5.448, *p* ≤ 0.01). Hypothesis 2 that postulated positive relationship between nutritional quality attributes and purchase intention of convenience food was accepted because standardized estimate (ß) of the path of structural model was significant (Hypothesis 2: ß = 0.639, S.E. = 0.056, *t*-value = 6.094, *p* ≤ 0.01). Hypothesis 3, which postulated positive relationship between safety attributes and purchase intention of convenience food, was accepted as the standardized estimate (ß) of the path of structural model was significant (Hypothesis 3: ß = 0.511, S.E. = 0.032, *t*-value = 16.063, *p* ≤ 0.01). Hypothesis 4 that proposed positive relationship between healthiness and purchase intention of convenience food was accepted, because the standardized estimate (ß) of the path of structural model was significant (Hypothesis 4: ß = 0.491, S.E. = 0.031, *t*-value = 15.594, *p* ≤ 0.01). Hypothesis 5, which postulated positive relationship between purchase intention and consumption of convenience food was accepted because standardized estimate (ß) of the path of structural model was significant (Hypothesis 5: ß = 0.998, S.E. = 0.016, *t*-value = 61.962, *p* ≤ 0.01). Further, Hypothesis 6 that proposed positive relationship between consumption and satisfaction towards convenience food was also accepted (Table 4) as standardized estimate (ß) of the path of structural model was statistically significant (Hypothesis 6: ß = 0.728, S.E. = 0.022, *t*-value = 32.516, *p* ≤ 0.01).

## 5. Discussion

The sensory appeal plays a significant role in driving consumers towards shopping and consumption of convenience food. The mean participants’ score of the sensory appeal construct and the standardized estimate of the path of structural model revealed that sensory appeal was the most important determinant influencing purchase intention, consumption, and satisfaction of consumers towards convenience food (Table 2 and Table 4). Further, the mean participants’ score of the items indicated that taste was the key factor influencing purchase intention, consumption, and satisfaction of consumers towards convenience food as compared to appearance, smell, and texture. Previous studies carried out under a wide range of social, cultural, and economic conditions also predicted sensory appeal as the most important determinant influencing shopping and consumption of convenience food [4,5,6,7,16,57]. Previous findings revealed that convenience, sensory appeal, nutritional quality, price, and health are important determinants influencing convenience food choice; however, the magnitude and importance of each determinant varied significantly across the social, cultural, economic and food related lifestyle [3,11,58].

In recent years, consumers have been more concerned about the quality and safety of convenience food. The development of novel and advanced food processing technologies such as high-pressure processing (HPP), pulse UV light, and irradiation has improved the quality of convenience food significantly [20,27]. The standardized estimate of the path of structural model indicated that nutritional quality attributes positively influenced purchase intention, consumption, and satisfaction of consumers towards convenience food (Table 4). The mean participants’ score indicated that food quality certification from a food regulatory agency was the most important among the factors under food nutritional quality construct, which drives consumers towards purchase intention and consumption of convenience food (Table 2). The findings of the previous studies also indicated that consumer perception towards quality attributes significantly influence purchase intention and consumption of convenience food [14,33,57]. Petrescu et al. [59] revealed that Belgian and Romanian consumers assign high values to quality attributes and often use taste, appearance, and freshness as an indicator to assess the quality of convenience food. The present findings also indicated that taste, appearance, and smells were key factors influencing convenience food choice, but these factors were considered under sensory appeal of convenience food (Table 2).

Food safety is another important aspect of convenience food that is directly associated with public health, food security, environmental protection, and sustainable development. The analysis of the structural model demonstrated that the safety attribute was positively associated with purchase intention, consumption, and satisfaction of consumers towards convenience food (Table 4). The mean participants’ score revealed that food safety certification was the most important factor under the food safety construct which drives consumers towards purchase and consumption of convenience food (Table 2). Previous studies also reported that food safety is an influential factor, which drives consumers towards convenience food choice [21,22,23,26]. The novel food processing technologies, i.e., HPP, pulse UV light, and irradiation, could be utilized by food processing industries in the production process to improve food quality and safety standards of convenience food [20,27].

The health benefit greatly influenced consumers towards convenience food choice. Due to technological development in processing, preservation, storage, and marketing, the sensory appeal, nutritional quality, safety, and health attributes of convenience food have been improved significantly in recent years [20,27]. The results of the structural model demonstrated the positive association between healthiness and purchase intention of convenience food (Table 4). The mean participants’ score of the health construct as well as different items within the health construct revealed that consumers are satisfied with the healthiness of convenience food. The overall results of the present study showed that the convenience food products are perceived as healthy and their consumption does not pose any threat to health (Table 2). This is due to the fact that the consumption of convenience food is not excessive, therefore the consumers have not reported any diverse effect of consuming convenience food on health. In contrast, consumers in developed and industrialized countries believe that consumption of convenience food has implication on health, diet quality, obesity, and chronic disease risk [32]. Vita et al. [57] revealed that high salt content, high fat content, and presence of nitrites had a negative impact on purchase intention of processed ham, but good taste, pleasant color, and juiciness diminishes the effect of aforementioned unhealthy compounds, which strongly support the findings of the present study in which consumers assign high values to sensory attributes as compared with quality, safety, and health attributes of convenience food.

In recent years, convenience food has spread into the lifestyle of consumers in emerging economies such as India due to time scarcity, competitive environment, and significant changes in food-related lifestyle [1,2]. Food production, processing, distribution, consumption, and waste disposal contribute largely to emission of greenhouse gasses, resources depletion, global warming, and environmental degradation [60,61,62]. Hence, environmentally sustainable food production, distribution, and consumption is important for sustainable development. Environmentally sustainable food consumption is the foremost important step to minimize the use of natural resources and emissions of greenhouse gasses, toxic waste, and pollutants, which in-turn enhance sustainable development and quality of life [63,64,65]. Convenience food involves production and transportation of raw materials, pre-processing manufacturing, packaging, distribution, consumption, and waste disposal which can be optimized in order to minimize the environmental degradation [66]. Food consumption behavior of consumers is a complex process and is strongly associated with lifestyle and socio-cultural environment. The consumers may express environmental concern, but during buying process normally ignore purchasing environmentally sustainable food products due to convenience, time pressure, availability, and price [67,68,69]. Previous studies carried out in developed and industrialized countries revealed that consumers should be encouraged to purchase environmentally sustainable convenience foods such as organic and minimally processed food to minimize negative effect on human health and environment [70]. Schmidt Rivera et al. [66] revealed that the environmental impact of ready to eat food was higher than equivalent home-cooked food. Further, consumers should be educated and encouraged to curtail ready-to-eat convenience food and consume more home-cooked food. In order to promote sustainable food consumption, consumers should be encouraged to purchase and consume plant-based foods because animal-based foods are more resource intensive and less environment friendly [71,72,73]. Environmental sustainability has become a severe concern, especially in developed and industrialized countries but in India, where the present study has been conducted, the consumers’ concern towards environmentally sustainable food production and consumption is insignificant. Sharma and Jha [74], in their study conducted in India, revealed that consumers’ individualism was negatively associated with sustainable food consumption. Government food regulatory bodies, non-government organizations, social and environmental activists, and policy makers should encourage and promote environment sustainable production, processing, distribution, and consumption of convenience foods in emerging economies such as India.

Though the present study provides in-depth knowledge and information regarding the role of sensory appeal, nutritional quality, safety, and health determinants on convenience food choice in emerging economies like India, the present study has some limitations. Due to time constraints, the study was carried in four cities in India which limits the generalization of the findings. Hence, future research should be carried out in different cities and countries in order to obtain more generalized and representative results. The present study concentrates on specific groups of consumers which also limits the applicability of the results. Therefore, future research should include wide range of consumers to improve overall applicability of the results. School children constitute an important consumer segment for convenience food. Hence, it is recommended to carry out similar studies for school children across cities and countries to provide them safe and healthy convenience food. Since environmental sustainability is a matter of grave concern, it is recommended to incorporate the environmentally sustainable aspects of convenience food in future studies in emerging economies, especially in India. For instance, packaging size of the convenience food would determine the amount of waste (packaging material after use) to be disposed of by a city. Similarly, it will influence the city’s environmental footprint in terms of waste collection and disposal. The findings of such studies would definitely enhance the knowledge and understanding about consumers’ purchase and consumption behavior towards convenience food in emerging markets.

## 6. Conclusions

The confirmatory factor analysis results indicated satisfactory and acceptable value of reliability of scale items and validity of the constructs of questionnaire. The model fit indices revealed that measurement and structural model relating sensory appeal, nutritional quality, safety attributes, and healthiness with purchase intention, consumption, and satisfaction of consumers towards convenience food were fitted well with data. Sensory appeal, quality attributes, safety attributes, and healthiness have a positive relationship with purchase intention, consumption, and satisfaction of consumers towards convenience food. Sensory appeal such as good taste, pleasant appearance, nice smells, and pleasant texture play the most important role in motivating and driving consumers towards purchase intention and consumption of convenience food. The overall result reveals that consumers give more importance to sensory appeal as compared with quality, safety, and health attributes during the purchase and consumption of convenience food in emerging economies such as India.

The conceptual framework and findings provide some theoretical and practical contributions. First, to the best of the authors’ knowledge, the present comprehensive study expands previous research by adding consumer satisfaction to a conceptual model relating sensory appeal, nutritional quality attributes, safety attributes, and healthiness with purchase intention, consumption, and satisfaction for convenience food. Second, the empirical evidence reveals that consumers in emerging economies assign high values to sensory appeal in convenience food choice, compared to quality, safety and health attributes, which shall add new information to literature. Third, the food processing industries should ensure that convenience foods are free from hormones, insecticides, pesticides, non-permissible additives, non-permissible colors, and artificial ingredients during production, processing, transportation, and marketing of convenience food to minimize health risk. Fourth, food processing industries should ensure the recommended level of calories, salt, sugar, and fat content in convenience food to provide a healthy and balanced diet to consumers. Finally, government food regulatory agencies should have strict food laws and regulations for mandatory food quality and safety certification to enhance consumers trust on convenience food.

## Figures and Tables

**Table 1:** Socio-demographic profile of participants.

Socio-Demographic Variables	Groups	Number of Participants	Percentage of Participants
Gender	Male	207	41.32
	Female	294	58.68
Age (years)	18–25	175	34.93
	26–35	203	40.52
	36–45	94	18.76
	46–65	29	5.79
Marital status	Single	245	48.90
	Married	256	51.10
Employment status	Unemployed	171	34.13
	Employed	330	65.87
Education level	10 *	2	0.40
	10 + 2 **	35	6.99
	Diploma	7	1.40
	Undergraduate	170	33.93
	Masters	173	34.53
	Doctoral	114	22.75
Annual family income (INR)	50,000–75,000	27	5.39
	75,000–100,000	32	6.39
	100,000–200,000	64	12.77
	200,000–500,000	140	27.94
	500,000–1,500,000	199	39.72
	1,500,000–3,000,000	37	7.39
	>3,000,000	2	0.40

*Note:* Total sample size = 501; 1 USD = INR 72. * 10 = high school; ** 10 + 2 = senior secondary school.

**Table 2:** Mean participants’ score, factor loading, Cronbach’s alpha(α), composite reliability (CR), and average variance extracted (AVE) of product determinants influencing purchase intention, consumption, and satisfaction of consumers for convenience food.

Construct	Items Code	Mean Score	Factor Loading	*p*- Value	α	CR	AVE
Sensory appeal (SEN)		4.12 ± 0.76			0.758	0.941	0.521
	SEN1	4.07 ± 0.64	0.778	***			
	SEN2	4.03 ± 0.63	0.779	***			
	SEN3	4.06 ± 0.62	0.704	***			
	SEN4	4.30 ± 0.63	0.613	***			
Nutritional quality (QUL)		3.87 ± 0.75			0.870	0.972	0.599
	QUL1	3.31 ± 1.04	0.812	***			
	QUL 2	3.16 ± 1.01	0.904	***			
	QUL3	3.16 ± 1.03	0.888	***			
	QUL4	3.66 ± 0.85	0.610	***			
	QUL5	3.52 ± 0.89	0.731	***			
	QUL6	3.80 ± 0.72	0.851	***			
Safety (SAF)		3.91 ± 0.68			0.897	0.979	0.566
	SAF1	3.55 ± 0.83	0.879	***			
	SAF2	3.63 ± 0.82	0.907	***			
	SAF3	3.61 ± 0.84	0.923	***			
	SAF4	3.61 ± 0.86	0.650	***			
	SAF5	3.61 ± 0.89	0.638	***			
	SAF6	3.48 ± 0.93	0.660	***			
	SAF7	3.83 ± 0.78	0.608	***			
Health (HEA)		3.71 ± 0.83			0.883	0.973	0.549
	HEA 1	3.02 ± 1.05	0.716	***			
	HEA2	2.88 ± 1.60	0.708	***			
	HEA3	3.17 ± 1.01	0.837	***			
	HEA4	3.45 ± 0.94	0.795	***			
	HEA5	3.52 ± 1.01	0.723	***			
	HEA6	3.38 ± 1.01	0.650	***			
Purchase intention (PI)		4.21 ± 0.91			0.780	0.900	0.576
	PI1	4.14 ± 0.81	0.628	***			
	PI2	4.17 ± 0.77	0.689	***			
	PI3	3.65 ± 1.03	0.842	***			
	PI4	3.59 ± 0.99	0.907	***			
	PI5	3.50 ± 1.03	0.754	***			
	PI6	4.20 ± 0.71	0.694	***			
	PI7	3.93 ± 0.91	0.763	***			
Consumption (CON)		3.95 ± 0.74			0.740	0.940	0.690
	CON1	3.83 ± 0.89	0.900				
	CON2	3.38 ± 0.89	0.767	***			
	CON3	3.79 ± 1.00	0.826	***			
	CON4	3.59 ± 0.65	0.765	***			
	CON5	3.81 ± 0.79	0.816	***			
	CON6	3.36 ± 0.74	0.912	***			
	CON7	3.67 ± 1.02	0.741	***			
Satisfaction (SAT)		4.20 ± 0.83			0.852	0.980	0.864
	SAT1	4.23 ± 0.64	0.891	***			
	SAT2	4.16 ± 0.63	0.879	***			
	SAT3	3.84 ± 0.71	0.927	***			
	SAT4	3.77 ± 0.83	0.938	***			
	SAT5	3.52 ± 0.91	0.913	***			
	SAT6	4.20 ± 0.62	0.905				
	SAT7	3.97 ± 0.66	0.963	***			
	SAT8	3.93 ± 0.66	0.948	***			
	SAT9	3.92 ± 0.67	0.962	***			
	SAT10	3.83 ± 0.74	0.940	***			
	SAT11	3.85 ± 0.70	0.952	***			
	SAT12	3.51 ± 0.86	0.898	***			

*** Significant at *p* ≤ 0.01; skewness: −1.067 to 0.322; kurtosis: −1.163 to 1.865. Note: See Appendix A for detailed description of the items. Measurement model fit indices: CFI = 0. 911; TLI = 0. 903; GFI = 0. 901; RMSEA = 0.072; SRMR = 0. 074.

**Table 3:** Discriminant validity of the constructs.

Constructs	Sensory Appeal	Nutritional Quality	Safety	Health	Purchase Intention
Sensory appeal	0.722				
Nutritional quality	0.243	0.774			
Safety	0.373	0.426	0.752		
Health	0.205	0.603	0.474	0.740	
Purchase Intention	0.184	0.552	0.425	0.624	0.758

**Table 4:** Structural model results to examine the association of between product determinants and purchase intention, consumption, and satisfaction for convenience food.

Hypothesis	Structural Path	Standardized Estimate (ß)	Standard Error (SE)	*t*-Value	*p*- Value	Results
H1	Sensory appeal → Purchase intention	0.788	0.053	5.448	***	Accepted
H2	Nutritional quality attribute → Purchase intention	0.639	0.056	6.094	***	Accepted
H3	Safety attribute → Purchase intention	0.511	0.032	16.063	***	Accepted
H4	Healthiness → Purchase intention	0.491	0.031	15.954	***	Accepted
H5	Purchase intention → Consumption	0.998	0.016	61.962	***	Accepted
H6	Consumption → Satisfaction	0.728	0.022	32.516	***	Accepted

*** Significant at *p* ≤ 0.01.

## Appendix A

Description of the questionnaire.

**Section 1**	**-**	**Socio-demographic characteristics**
Gender		
Age		
Marital status		
Employment status		
Education level		
Family income		
Food habits		
Food preferences		
Frequency of eating convenience food		
Health concern		
**Section 2**	**-**	**Sensory appeal (SEN)**
SEN1	-	I prefer convenience food because it has a pleasant appearance.
SEN2	-	I prefer convenience food because it smells nice.
SEN3	-	I prefer convenience food because it has pleasant texture.
SEN4	-	I prefer convenience because it tastes good.
**Section 3**	**-**	**Nutritional quality (QUL)**
QUL1	-	I prefer convenience food because of its high nutritive value.
QUL2	-	I prefer convenience food because it has high mineral content.
QUL3	-	I prefer convenience food because it has high vitamin content.
QUL4	-	I prefer convenience food because it contains natural ingredients.
QUL5	-	I prefer convenience food because it has high fiber content.
QUL6	-	I prefer convenience food because it has necessary quality certification.
**Section 4**	**-**	**Safety (SAF)**
SAF1	-	I prefer convenience food because it is free of hormones.
SAF2	-	I prefer convenience food because it is free of insecticides.
SAF3	-	I prefer convenience food because it is free of pesticides.
SAF4	-	I prefer convenience food because it doesn’t contain any non-permissible additives.
SAF5	-	I prefer convenience food because it doesn’t contain any non-permissible color.
SAF6	-	I prefer convenience food because it doesn’t contain any artificial ingredients.
SAF7	-	I prefer convenience food because it has necessary safety certification.
**Section 5**	**-**	**Health (HEA)**
HEA 1	-	I prefer convenience food because it has low calories.
HEA2	-	I prefer convenience food because it has low fat content.
HEA3	-	I prefer convenience food because it has low salt content.
HEA4	-	I prefer convenience food because it has low sugar content.
HEA5	-	I prefer convenience food because it provides me with a balanced diet.
HEA6	-	I prefer convenience food because I have more energy after consuming.
**Section 6**	**-**	**Purchase intention (PI)**
PI1	-	I will continue to buy convenience food due to competitive price and promo tional offer.
PI2	-	I will continue to buy convenience food to save time.
PI3	-	I will continue to buy convenience food due to lack of cooking skills and motivation.
PI4	-	I will continue to buy convenience food to reduce environmental damage
PI5	-	I will continue to buy convenience food due to good quality, safety, and health attributes.
PI6	-	I will continue buy convenience food because it is readily available and easy to Prepare.
PI7	-	I will continue to buy convenience food as there are choices available for multi cuisines.
**Section 7**	**-**	**Consumption (CON)**
CON1	-	I consume convenience food due to convenience.
CON2	-	I consume convenience food due to minimum physical and mental effort to cook.
CON3	-	I consume convenience food due to good taste, smell, and appearance.
CON4	-	I consume convenience food due to attractive packaging.
CON5	-	I consume convenience food due to competitive price.
CON6	-	I consume convenience food due to good quality, high safety, and healthiness.
CON7	-	I consume convenience food due to my religious and ethical beliefs.
**Section 8**	**-**	**Satisfaction (SAT)**
SAT1	-	I am satisfied with time saving.
SAT2	-	I am satisfied with easy cooking.
SAT3	-	I am satisfied with easy storage.
SAT4	-	I am satisfied with the availability.
SAT5	-	I am satisfied with price.
SAT6	-	I am satisfied with taste.
SAT7	-	I am satisfied with the appearance.
SAT8	-	I am satisfied with the smell.
SAT9	-	I am satisfied with texture.
SAT10	-	I am satisfied with quality attributes.
SAT11	-	I am satisfied with safety attributes.
SAT12	-	I am satisfied with the health issues.
